# Shotgun metagenomic analysis reveals new insights into bacterial community profiles in tempeh

**DOI:** 10.1186/s13104-020-05406-6

**Published:** 2020-12-11

**Authors:** Adi Yulandi, Antonius Suwanto, Diana Elizabeth Waturangi, Aris Tri Wahyudi

**Affiliations:** 1 Department of Biology, Faculty of Mathematics and Natural Science, IPB University (Bogor Agricultural University), Gedung Biologi. Jalan Agatis Kampus IPB Dramaga, 16680 Bogor, Indonesia; 2 Faculty of Biotechnology, Atma Jaya Catholic University of Indonesia, Jalan Jenderal Sudirman 51, 12930 Jakarta, Indonesia

**Keywords:** Tempeh, Shotgun metagenomic, *Proteobacteria*, *Firmicutes*

## Abstract

**Objective:**

Amplicon sequencing targeting 16S ribosomal RNA (rRNA) has been widely used to profile the microbial community from fermented food samples. However, polymerase chain reaction (PCR) steps on amplicon sequencing analysis and intragenomic heterogeneity within 16S rRNA are believed to contribute to bias in estimating microbial community composition. As potential paraprobiotics sources, a comprehensive profiling study of tempeh microbial ecology could contribute to tempeh product development. This study employed a shotgun metagenomic approach, where metagenome fragments from tempeh samples were sequenced directly for taxonomic and functional profiling analysis.

**Results:**

Taxonomic profiling showed that *Proteobacteria*, *Firmicutes*, and *Bacteroidetes* were the dominant phyla from the shotgun metagenomic analysis in all tempeh samples. In terms of composition, this shotgun metagenomic study revealed that *Proteobacteria* was the most abundant phylum. Functional profiling showed that iron complex outer-membrane recepter protein (KEGG ID: K02014) was the most transcribed gene based on this metagenomic analysis. The metagenome-assembled genomes (MAGs) results from the binning pipeline could reveal almost complete whole genome sequence of *Lactobacillus fermentum*, *Enterococcus cecorum*, *Escherichia coli*, *Klebsiella pneumoniae*, and *Acinetobacter baumannii*.

## Introduction

Tempeh is fermented food originated from Indonesia. The biochemical changes of soybean during microbial fermentation increased nutritional values and health-promoting bioactive compounds in tempeh. Compared to other indigenous soybean-based fermented food such as *nato*, *miso* (Japan), *kinema* (Nepal), and *douchi* (China), which used *Bacillus* spp. as inoculum, tempeh used *Rhizopus* spp. in the production [1]. The nature of tempeh production processes creates consortia of microorganisms not only from tempeh inoculum but also from production materials and environment [2]. Over the past decade, useful tools of next-generation sequencing (NGS) such as metagenomics has been applied to study microbial consortia from fermented food microbial ecology [3]. Previous metagenomic studies of the microbial community during tempeh production were conducted by employing amplicon sequencing targeting the V4 region of the 16S rRNA gene. These studies focused on the dynamic taxonomic profile of the microbial community from tempeh metagenome samples and indicated that *Firmicutes* was the predominant phylum. Some genera such as *Lactobacillus*, *Streptococcus*, and *Weisella* from *Firmicutes* phylum known as probiotics are also reported in this study [4–6]. Tempeh is generally cooked before consumption and might act as an inactivated probiotics source. Previous tempeh nutrigenomic studies showed that cooked tempeh supplementation could enhance the immune system by increasing IgA production in human intestinal tracts [7]. Recently, studies reported cooked tempeh consumption for 6 months, improving global cognitive function respondents aged 60 years or over with mild cognitive impairment [8]. Inactivated probiotics were previously mentioned in literature with the term paraprobiotics. Recently paraprobiotics are defined as non-viable either intact or broken cells of microbial and crude extract of cells that could positively affect when administered in a sufficient amount [9]. There are around 80,000 tempeh producers categorized as small and medium-scale home industries in Indonesia [10]. The tempeh microbial ecology formed during production may vary among these producers. As potential paraprobiotics sources, a comprehensive profiling study of tempeh microbial ecology could contribute to tempeh product development. Besides for taxonomic profile and composition study, shotgun metagenomics could also be used for the functional study of microbial communities based on more objective analysis through direct whole-genome sequencing [11, 12]. Therefore, this study aims to study tempeh microbial ecology employing shotgun metagenomic analysis.

## Main text

### Materials and methods

#### Samples

Samples were collected from two local traditional tempeh producers in Bogor, Indonesia, designated as EMP and WJB. The samples from these producers have been used as sources for microbial community analysis on tempeh for many years [5]. EMP and WJB were representatives for the different methods of soybean boiling process on tempeh productions. The EMP employs one boiling process, while the WJB employs two boiling processes. Five tempeh samples from the same production batch were randomly picked from the EMP or WJB producers. The plastic was used as a wrapper in the tempeh production process. On the same day, each of these tempeh samples small diced and pooled in a sterile container for further step, total microbial DNA genome extraction.

#### Total DNA extraction

The extraction process was adapted from a previous study [13]. One hundred-gram of fresh tempeh sample was homogenized in 300 mL of phosphate buffer saline (PBS) using the Philip HR2061 blender (Koninklijke Philips, Amsterdam, Netherland) for 30 s. The homogenate was centrifuged at 1000×*g* for 10 min. Supernatants were collected and centrifuged at 10,000×*g* for 3 min. The pellets were subjected to total microbial DNA extraction employing ZymoBIOMICS DNA/RNA Mini Kit (Zymo Research, California, USA) protocols.

#### Metagenome sequencing

The whole metagenome library preparation and sequencing process used services from NovogeneAIT Genomics Singapore Pte Ltd. The whole microbial DNA was sheared to produce fragment libraries using restriction enzymes with a minimum of 1 µg of DNA as input. Microbial DNA was precisely quantified using Qubit 2.0 (Thermo Fischer Scientific, United States). The purity and degradation assessment for microbial DNA was done employing NanoDrop (Thermo Fischer Scientific, United States) and gel electrophoresis. The paired-end sequencing library was prepared using the TruSeq DNA PCR-Free Prep Kit (Illumina, United States). The prepared library was sequenced on the NovaSeq 6000 platform (2 × 150 bp chemistry) (Illumina, United States).

#### Shotgun metagenomic sequencing data analysis

The sequenced reads (raw reads) were filtered from reads containing adapters, reads containing N (the base cannot be determined) > 10%, and reads containing low-quality (Qscore ≤ 5) base, which is over 50% of the total base, by using NovogeneAIT Genomics Singapore Pte Ltd pipeline to produce high-quality paired-end reads (clean reads). *Rhizopus* spp. reads contamination was removed from the clean reads by employing Read QC module from MetaWRAP pipeline [14] using the mix *R oryzae* 99892 (PRJNA186020), *R. microsporus* ATCC 52813 (PRJNA430271, PRJNA205957), *R. delemar* RA 99-880 (PRJNA13066), *R. stolonifer* B9770 (PRJNA184886) and *R. azygosporus* (PRJNA418064) whole-genome sequence as a reference. The SqueezeMeta pipelines [15] were employed for assembly, taxonomic, functional, and bin analyses. The pipelines used the co-assembly mode option where reads from all samples were pooled before the assembly using the Megahit [16] step was performed. The SQMtools package on R version 4.0.3 [17] was used to analyze both taxonomic and functional profiling data generated from SqueezeMeta pipelines [18].

### Result

#### Metagenome sequencing and assembly statistic

The total microbial DNA extracted from tempeh samples collected from two different tempeh producers in Bogor, Indonesia were subjected to Illumina whole metagenome sequencing pipelines. The average effective rate of clean reads from two raw reads of metagenomic data after the quality trimming was 99.93%. A total of 29,030,144 (36.74%) reads from 78,995,980 EMP clean reads and 17,146,676 (15.86%) reads from 108,045,092 WJB clean reads were mapped to the *Rhizopus* spp. genome reference. The number of contigs resulting from the co-assembly steep data was 293,961. The longest contigs were 485,167 bp, and the N50 value was 1994.

#### Taxonomic and functional profiling

**Fig. 1:**
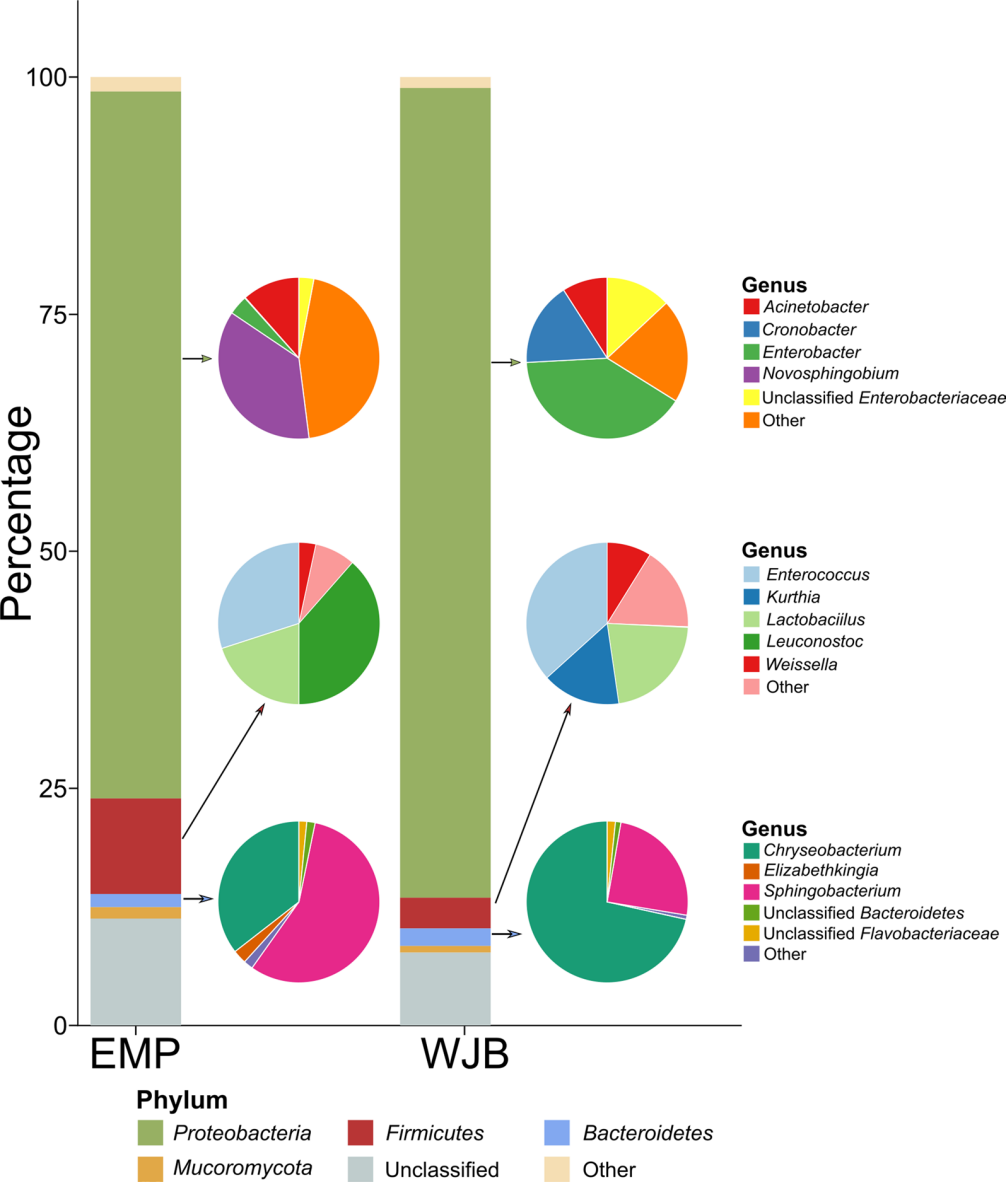
The taxonomic abundance of the microbial community of tempeh samples at the rank of phylum and genus

**Fig. 2:**
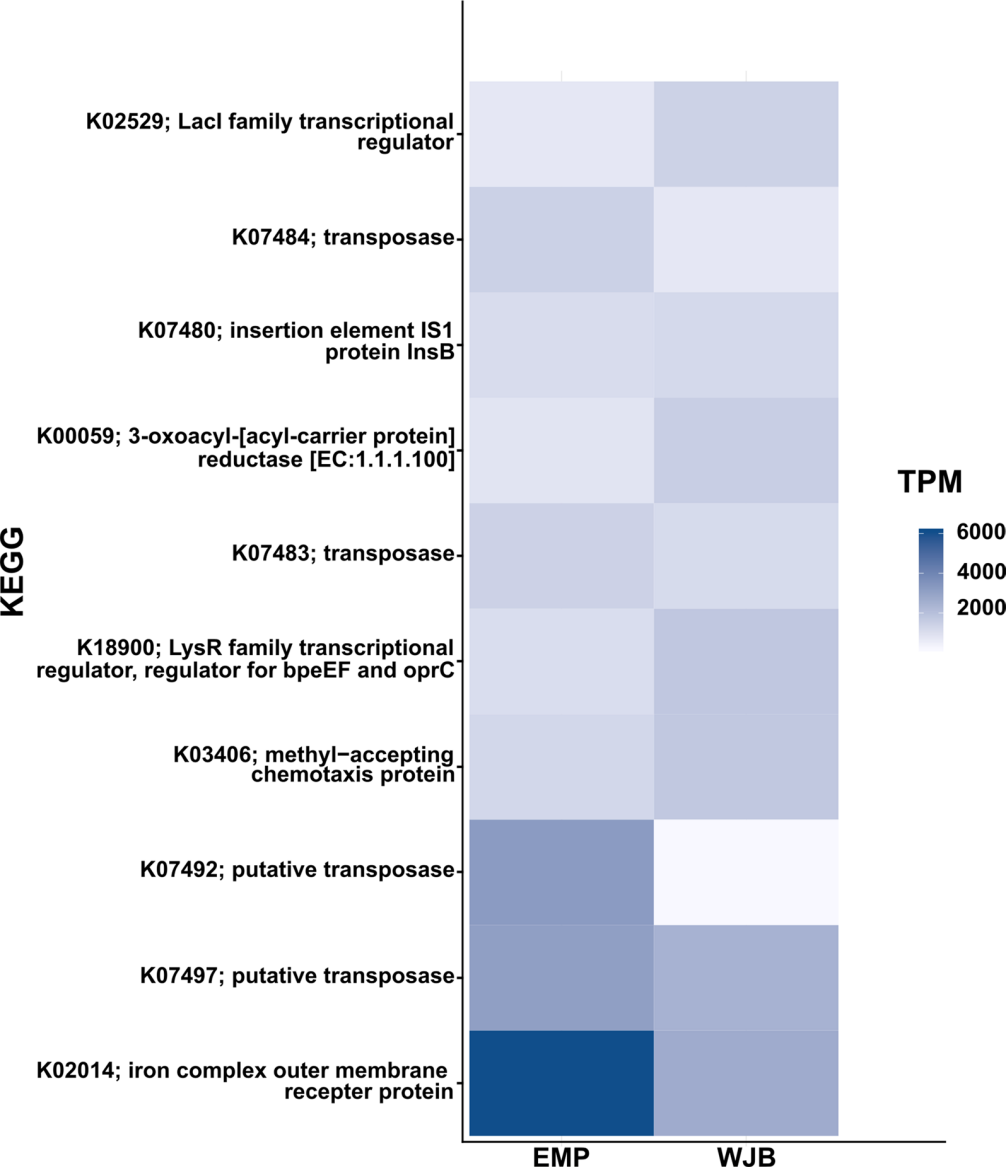
The functional profile of the tempeh metagenome samples using KEGG annotation in TPM (transcripts per kilobase million)

The taxonomic assignment of contigs is based on individual genes taxonomic assignment. The SqueezeMeta pipeline implements a fast-last common ancestor (LCA) algorithm to analyze each query gene hit results as the Diamond [19] search query against the GenBank nr database. The contigs are annotated to a consensus of the taxon to which most of their genes belong. The selected hits must pass a minimum amino acid identity (AAI) level for assignment to taxonomic ranks. For the phylum and genus, the threshold was 40% and 60% [20]. The metagenome reads will map onto contigs using Bowtie2 [21] to estimate each gene and contig abundance. Contigs with phylum annotation were 263,096 (89.5%). *Proteobacteria* was a relatively abundant phylum in EMP (74.54%) and WJB (85.38%) metagenome. In the genus level, 211,603 (72%) contig were annotated. *Novosphingobium* was a relatively abundant genus in the EMP metagenome (27.16%) and the *Proteobacteria* phylum of EMP (26.81%). *Enterobacter* was a relatively abundant genus in the WJB metagenome (34.39%) and the *Proteobacteria* phylum of WJB (33.93%). *Firmicutes* phylum in the EMP metagenome (10.07%) was relatively more abundant than the WJB metagenome (3.23%). *Leuconostoc* (3.83%), *Enterococcus* (2.99%), and *Lactobacillus* (1.98%) were the top three genera in the *Firmicutes* phylum of EMP. In the *Firmicutes* phylum of WJB, *Enterococcus* (1,17%) was the most abundant genus. The abundance of *Bacteroidetes* phylum was relatively similar in EMP (1.38%) and WJB (1.84%) metagenome samples (Fig. 1). Functional profiling used the latest publicly available version of the KEGG database for KEGG ID annotation. Iron complex outer membrane recepter protein (KEGG ID: K02014) was the most transcripted expression in the metagenome from tempeh samples (Fig. 2).

#### Binning and bin check

The total number of bins obtained from the co-assembly of EMP and WJB metagenome samples results from the DAS tool [22] was 25. According to the CheckM [23] result, eleven bins were categorized as good-quality bins, whose completeness was more than 75% with less than 10% contamination (Table 1). Among good-quality bins, four bins were categorized as high-quality bins, whose completeness was more than 90%.

**Table 1:** Good-quality bins (> 75% completion, < 10% contamination) obtained by co-assembly mode of EMP and WJB metagenome

Taxa	Size (bp)	Completeness (%)	Contamination (%)	Taxonomic rank
*Lactobacillus fermentum*	1,873,792	92.73	3.12	Species
*Enterococcus cecorum*	2,275,479	88.56	2.99	Species
*Escherichia coli*	4,443,894	84.94	3.56	Species
*Klebsiella pneumoniae*	4,774,975	84.5	9.37	Species
*Acinetobacter baumannii*	2,430,198	79.08	2.36	Species
*Proteobacteria*	2,219,482	88.31	8.82	Phylum
*Proteobacteria*	3,636,366	79.83	4.62	Phylum
*Bacteria*	5,137,284	100	1.82	Kingdom
*Bacteria*	4,249,945	91.22	1.72	Kingdom
*Bacteria*	6,839,222	90.75	3.61	Kingdom
*Bacteria*	1,975,733	79.31	0.86	Kingdom

## Discussion

The relative abundance of each gene and contigs on the shotgun metagenomic data required normalization because of the genome length differences. The SqueezeMeta pipeline develops a custom script to compute the normalization gene and contig abundance [15]. Study of the microbial community of EMP and WJB tempeh samples using metagenomic16S rRNA sequencing analysis [4–6] is consistent with the result of shotgun metagenome sequencing analysis in this study, i.e., *Proteobacteria*, *Firmicutes*, and *Bacteroidetes* were the three most abundant phyla. For the EMP and WJB tempeh sample, *Firmicutes* were reported as the most abundant phylum in the previous amplicon metagenomic study [4, 5]. In contrast, in this study, the most abundant phylum was *Proteobacteria*. The previous amplicon metagenomic study on EMP and WJB sample revealed the second-times soybean boiling process on the WJB tempeh production might contribute to tempeh microbial community profile [5]*. Firmicutes* phylum on the EMP (92%) sample reported relatively more abundant compare to the WJB (88%). This similar trend was also reported in this study. At the genus level from this phylum, both metagenomic analyses revealed *Enterococcus* relatively most abundant genus in the EMP samples. In the WJB samples, this study also reports *Enteroccous* was the relatively most abundant genus, while in amplicon metagenomic study was genus *Lactobacillus*. In terms of taxa prediction and abundance estimation, a recent study reported shotgun metagenomic analysis produced more accurate results compared to 16S rRNA gene-based metagenomic analysis from an artificial skin-associated microbial community. This artificial microbial community contains four species from phylum *Proteobacteria*, eight species from phylum *Firmicutes*, and three species from phylum *Actinobacteria* [24]. Taxonomic profiling solely based on the 16S rRNA sequence could generate bias in bacterial cell count from metagenome samples because many bacteria possess more than one different copy number of this gene [25]. Different PCR primers used on PCR protocols preferentially amplify different taxa sets and generate bias for amplicon metagenomic analysis [26]. A study on the bacterial community in EMP tempeh employing culturable technique also reported that *Proteobacteria* was the most dominant phylum [5]. The functional profiling of tempeh metagenome samples showed that the most annotated functions were transporter and transposase related genes. It has been reported that the bioavailability of minerals such as iron during tempeh production was significantly elevated. Tempeh has been known to reduce the level of chelating agents, such as phytic acid typically present in soybean [27]. For most bacteria, iron is an essential micronutrient. Gram-negative bacteria such as species in phylum *Proteobacteria* use the classical iron transport system Iron complex outer-membrane recepter protein (KEGG ID: K02014) is part of it [28]. Previous study demonstrated that transposase was the most abundant gene in environmental metagenome. Transposase catalyzes `cut-and-paste’ or ‘copy-and-paste’ reactions promoting DNA segments mobility of to new sites. Transposase may mobilize or activate genes that enhance their hosts fitness [29]. The binning pipeline was able to produce good quality (> 75% completion and < 10% contamination) the metagenome-assembled genomes (MAGs). Among these MAGs, five were in the species level of taxonomic rank. Some *K. pneumoniae* isolates from EMP and WJB tempeh fully sequenced and subjected to genomic comparison to pathogenic strain in the previous study [30]. *K. pneumoniae* is known to produce vitamin B_12_ during tempeh fermentation [31]. Data analysis from the amplicon sequencing of metagenomic samples of EMP and WJB tempeh in the previous study [4, 5] failed to reveal this important genus. The binning generated draft genome of *Lactobacillus fermentum*. Previous study showed that lactic acid bacteria (LAB) were common microbial communities found during tempeh production [32]. Genetic diversity employing enterobacterial repetitive intergenic consensus-polymerase chain reaction (ERIC-PCR) of *E. coli* isolated from tempeh samples were reported [33]. This study showed that *E. coli* from tempeh samples are genetically different from medical isolates. Partial complete of *E. coli* genome draft in this study will significantly contribute further comparative genomic study, especially for *E. coli* isolates derived from tempeh. Beneficials tempeh supplementation as a paraprobiotics source has been published [7, 8], it is reported the whole non-viable might affect human immune and cognitive system. Detail contribution from the tempeh microbial community has not yet been explored. The knowledge from this study might enhance tempeh as a potential paraprobiotics source development.

## Limitation

This study design only employed samples from two producers that could generate bias of taxonomic and functional profiling. While we did not analyze blank controls in this study, we utilized a sterile technique to minimize the effects of laboratory contamination.

## Data Availability

The tempeh metagenomic raw reads used for this study were deposed in publicly accessible NCBI’s Sequence Read Archive (SRA) under the accession number: PRJNA605305 (https://www.ncbi.nlm.nih.gov/bioproject/PRJNA605305/).

## Abbreviations

rRNA: Ribosomal RNA
PCR: Polymerase Chain Reaction
MAGs: The metagenome-assembled genomes
NGS: Next-Generation Sequencing
PBS: Phosphate Buffer Saline
LCA: Last Common Ancestor
AAI: Amino acid identity
KEGG: Kyoto Encyclopedia of Genes and Genomes
LAB: Lactic acid bacteria
ERIC-PCR: Enterobacterial repetitive intergenic consensus-polymerase chain reaction
TPM: Transcripts per kilobase million
